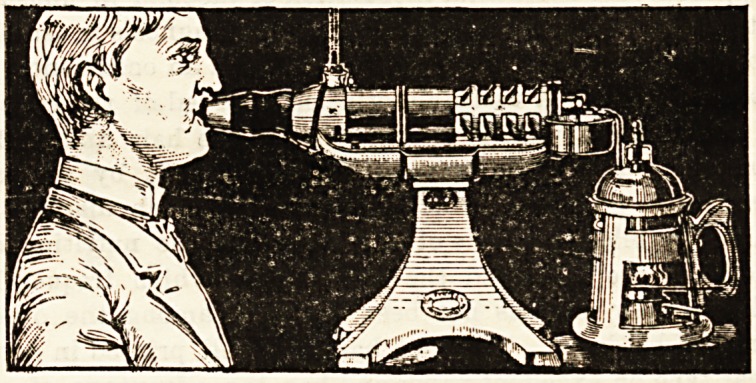# New Appliances and Things Medical

**Published:** 1904-10-08

**Authors:** 


### NEW APPLIANCES AND THINGS MEDICAL.

### THE "THERMO-VARIATOR."

A New Regulating Inhaler.

Inhalation has long occupied a prominent position among methods of treating affections of the respiratory organs. Inhalers of all sorts and conditions have been introduced, and to the many has now been added another, the "Thermo-Variator" of Dr. Bulling (English Agents: F. Krasa and Co., 7 Wood Street Square, London, E.C.). The apparatus is elaborate and somewhat expensive, but after a careful examination and thorough testing we are of opinion that this inhaler will prove of considerable advantage. The apparatus consists of the atomiser, composed of a boiler with safety-valve and steam-pipe, and cup for the medicament, and the inhaling tube composed of the mouth or nosepiece, a conic tube covered by a metal cylinder which allows of the regulation of the amount of air mixing with the medicated steam, and the supporting vessel which is made of fine China-clay. The principal parts of the apparatus are all made of China-clay, and thus offer many hygienic advantages, but, the component parts being readily breakable, much care must be exercised in the use of the inhaler. The apparatus may also be used for rendering the atmosphere of a room humid or making it actually medicated. A cursory glance at this somewhat cumbrous and not altogether attractive appliance will probably arouse the scorn of cynics, but after personal trial of the inhaler we recommend it to the unprejudiced consideration of the profession.

## Figures and Tables

**Figure f1:**